# A Systematic Review Evaluating the Effectiveness of Several Biological Therapies for the Treatment of Skin Psoriasis

**DOI:** 10.7759/cureus.50588

**Published:** 2023-12-15

**Authors:** Sattam A Alzahrani, Fajer M Alzamil, Abdulaziz M Aljuhni, Naif A Al Thaqfan, Norah Y Alqahtani, Sara A Alwarwari, Abdullah A Alkharashi, Rakan A Alzabadin, Reema A Alzehairi, Abdullah A Alhajlah

**Affiliations:** 1 General Practice, Medical Graduate of Al-Imam Mohammed Bin Saud Islamic University, Riyadh, SAU; 2 Dermatology, College of Medicine, Imam Mohammed Bin Saud Islamic University, Riyadh, SAU; 3 General Practice, Graduate of Princess Nourah Bint Abdulrahman University, Riyadh, SAU; 4 General Practice, Medical Graduate of Vision Colleges, Riyadh, SAU; 5 Dermatology, King Saud Bin Abdulaziz University for Health Sciences College of Medicine, Riyadh, SAU

**Keywords:** biologic treatment, dermatology, effectiveness, biological therapies, psoriasis

## Abstract

Psoriasis is a chronic inflammatory skin illness that has the potential to manifest at any stage of life, it is most frequently observed in early adulthood. Biological drugs have significantly transformed the landscape of psoriasis treatment through the provision of focused therapy, which effectively mitigates inflammation and regulates the overproduction of skin cells. Notwithstanding the accessibility of these biological drugs, rigorous evaluations that juxtapose their safety and efficacy profiles are necessary. The objective of this study is to conduct a thorough investigation of the relative efficacy of these drugs in alleviating psoriasis symptoms and increasing the quality of life for patients by synthesizing the existing evidence. A comprehensive review was conducted to evaluate and compare the safety and effectiveness of different biochemical medicines utilized in the management of psoriasis. In accordance with the Preferred Reporting Items for Systematic Reviews and Meta-Analyses (PRISMA) recommendations, the review process was conducted among the available studies. A search was conducted across electronic databases, such as Web of Science, PubMed, and Embase, utilizing a combination of keywords and Mesh phrases pertaining to psoriasis, biological medications, and particular names of pharmaceuticals.

In total, 475 studies were ascertained by the preliminary search of the database. After eliminating duplicate research, 358 distinct studies remained. After meticulous screening of titles and abstracts against the predefined inclusion criteria, 281 papers were deemed ineligible and thus excluded. For final inclusion, the whole texts of the remaining 77 studies were evaluated. Forty additional papers were removed during the full-text evaluation for a variety of reasons, including improper research design, or insufficient outcome data. Finally, 37 studies were included in this systematic review since they satisfied all inclusion criteria. The results of the current systematic review showed that all biological medications showed high efficacy in the treatment of skin psoriasis compared with placebo based on the clinical assessment outcomes using different tools such as PASI.

## Introduction and background

Psoriasis is a chronic inflammatory skin illness characterized by a fast accumulation of skin cells, culminating in thick, scaly plaques [1]. It can cause substantial physical and psychological anguish and impacts millions of individuals globally [2,3]. Psoriasis’s precise etiology remains uncertain; nevertheless, it is hypothesized to be the result of an intricate interplay between environmental and genetic influences [4,5].

Psoriasis has considerable variation in prevalence across distinct populations, with global estimates spanning from 0.1% to 3% [6]. Although it has the potential to manifest at any stage of life, it is most frequently observed in early adulthood [7]. In addition to nails, psoriasis can impact the scalp, elbows, and knees, among other body areas [8].

Biological drugs have significantly transformed the landscape of psoriasis treatment through the provision of focused therapy, which effectively mitigates inflammation and regulates the overproduction of skin cells. Having demonstrated exceptional effectiveness in clinical studies, they have received approval for the treatment of psoriasis. Risankizumab, secukinumab, guselkumab, adalimumab, certolizumab, etanercept, ustekinumab, brodalumab, ixekizumab, tildrakizumab, infliximab, methotrexate, briakinumab, golimumab, and adalimumab are some examples of biological medicines frequently used in the management of psoriasis [9,10].

Biological therapies are advised for the treatment of psoriatic disease in all six domains of the disease [11]. The primary aim in the therapy of psoriasis is to establish a comprehensive, safe, and efficacious treatment regimen that addresses all of its manifestations [12]. Nevertheless, the attainment of this objective is complicated by the diversity of the manifestations. Recent developments in our understanding of the disease's pathogenesis have prompted substantial research and approval of various modes of action, including TNFi (INFLIXIMAB, etanercept, golimumab, certolizumab, and adalimumab); IL-17i (secukinumab, ixekizumab, and brodalumab); and IL-12 and/or IL23i (ustekinumab, guselkumab, Risankizumab, and tildrakizumab).

These pharmaceuticals function via distinct methods of action. As an illustration, ixekizumab, Risankizumab, secukinumab, and guselkumab selectively target interleukin-17A (IL-17A), a protein that is pivotal in the inflammatory mechanism underlying psoriasis [10]. Through the inhibition of IL-17A, these pharmaceutical agents aid in the mitigation of inflammation and amelioration of symptoms [10]. Additional biological drugs, including infliximab, adalimumab, certolizumab, and etanercept, selectively interact with tumor necrosis factor-alpha (TNF-alpha), a molecule that is implicated in the immune response associated with psoriasis [13]. By suppressing TNF-alpha, these drugs aid in illness management and inflammation relief [13].

Ustekinumab selectively inhibits interleukin-12 (IL-12) and interleukin-23 (IL-23), cytokines that play a role in the psoriasis immune response [14]. Through the inhibition of IL-12 and IL-23, ustekinumab aids in inflammation reduction and immune system regulation [14]. Alternative pharmaceuticals, including methotrexate, briakinumab, golimumab, and ADA (adalimumab), operate by means of distinct pathways and selectively target distinct immune system components in order to elicit therapeutic responses [15].

Notwithstanding the accessibility of these biological drugs, rigorous evaluations that juxtapose their safety and efficacy profiles are necessary. These studies offer significant insights regarding the relative efficacy of various treatments and serve as a reference for clinical decision-making. Nevertheless, the number of systematic reviews and comparisons of these biological medicines for the treatment of psoriasis is insufficient.

As a result, by a systematic evaluation of the existing literature concerning the efficacy of several biological medicines for the treatment of psoriasis, this study seeks to fill this knowledge gap. The objective of this study is to conduct a thorough investigation of the relative efficacy of these drugs in alleviating psoriasis symptoms, decreasing inflammation, and increasing the quality of life for patients by synthesizing the existing evidence. The outcomes of this research results will augment the existing body of knowledge regarding the management of psoriasis and provide guidance to medical practitioners in the process of prescribing the most suitable biological therapy for their clients.

## Review

Methodology

Study Design

A comprehensive review was undertaken to evaluate and compare the effectiveness of diverse biological medicines utilised in the management of psoriasis. In accordance with the Preferred Reporting Items for Systematic Reviews and Meta-Analyses (PRISMA) recommendations, the review process was conducted with integrity and transparency.

Search Strategy

A thorough examination of the literature was undertaken in order to locate pertinent studies. A search was conducted throughout electronic databases, such as Web of Science, PubMed, and Embase. The search strategy involved a meticulous combination of keywords and MeSH phrases related to psoriasis, biological medications, and specific pharmaceuticals. The keywords used in this research including “Psoriasis”, “biological treatment”, “TNF-alpha inhibitor”, “IL-12/IL-23 Inhibitors”, ” IL-17 Inhibitors”, “IL-23 Inhibitors”, “IL-23/IL-17 Inhibitors”, “Risankizumab”, “secukinumab”, “guselkumab”, “adalimumab”, “certolizumab”, “etanercept”, “ustekinumab”, “brodalumab”, “ixekizumab”, “tildrakizumab”, “Infliximab”, “methotrexate”, “briakinumab”, “golimumab”, “bimekizumab” and “ADA”. The search methodology was modified in accordance with the specific criteria of every database.

Study Selection Criteria

The following inclusion criteria were applied to identify eligible studies.

Participants: Studies involving patients diagnosed with psoriasis, including both plaque psoriasis.

Intervention: Randomized controlled trials (RCTs) evaluating the use of biological medications (Risankizumab, secukinumab, guselkumab, adalimumab, certolizumab, etanercept, ustekinumab, brodalumab, ixekizumab, tildrakizumab, infliximab, methotrexate, briakinumab, golimumab, bimekizumab and ADA) for the treatment of psoriasis.

Comparator: Studies comparing the efficacy of different biological medications or comparing biological medications with placebo or other standard treatments.

Outcome measures: Studies reporting outcomes related to disease severity, such as Psoriasis Area and Severity Index (PASI) scores.

Study Design

Only RCTs were included in this review.

Study Selection Process

Five independent reviewers independently screened the titles and abstracts of the identified studies to assess their eligibility based on the inclusion criteria. Full-text articles of potentially eligible studies were obtained and assessed for final inclusion. Any discrepancies or disagreements between reviewers were resolved through discussion or consultation with a sixth or seventh reviewer if necessary.

Data Extraction

Data extraction was performed independently by five reviewers using a standardized data extraction form. The following information was extracted from each included study: study characteristics (authors, year of publication, study design), participant characteristics (sample size, demographics), intervention details (type of biological medication, dosage, duration of treatment), comparator details, outcome measures, and results.

Results

**Figure 1:**
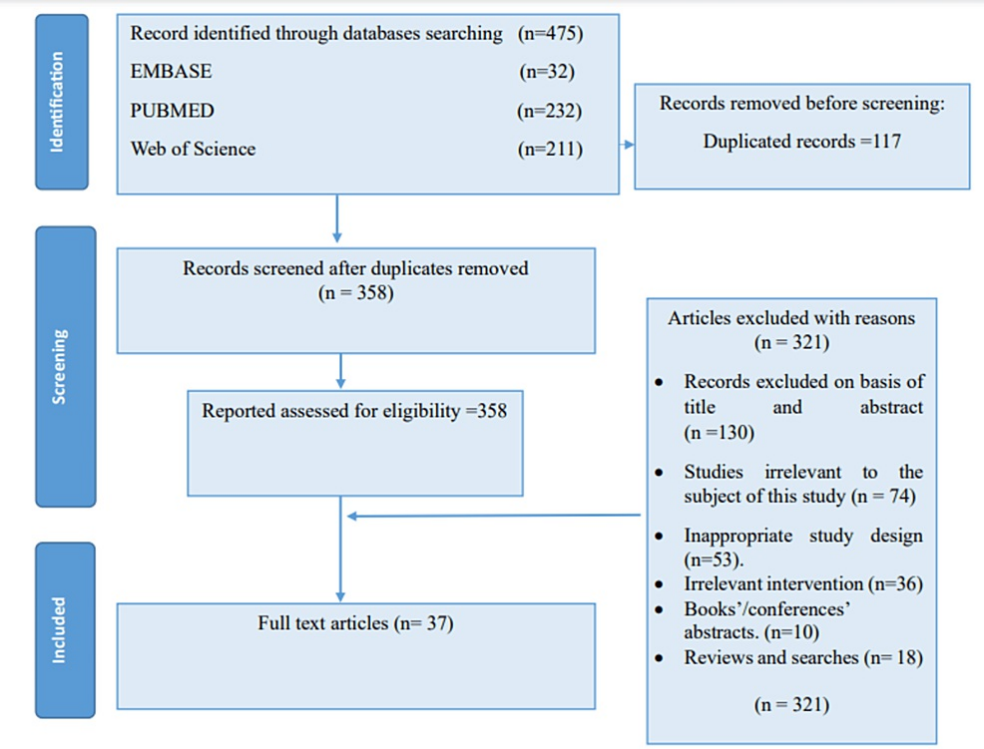
The PRISMA figure showing the steps to choose the studies for systematic review PRISMA: Preferred Reporting Items for Systematic Reviews and Meta-Analyses

In total, 475 studies were ascertained by the preliminary search of the database. After eliminating duplicate research, 358 distinct studies remained. After meticulous screening of titles and abstracts against the predefined inclusion criteria, 281 papers were deemed ineligible and thus excluded. For final inclusion, the whole texts of the remaining 77 studies were evaluated. Forty additional papers were removed during the full-text evaluation for a variety of reasons, including improper research design, irrelevant intervention, or insufficient outcome data. Finally, 37 studies were included in this systematic review since they satisfied all inclusion criteria. A detailed flowchart with the results of the literature review is shown in Figure 1.

For the skin domain, results between 10 and 16 weeks were considered which is reported in all studies. The systematic review included a total of 37 RCTs focusing on the outcomes of the GRAPPA domain. These trials investigated various drugs and dosages and assessed multiple outcomes related to psoriasis. The review encompassed studies conducted between 2005 and 2021, with sample sizes ranging from 78 to 1,881 participants. The drugs evaluated in the trials included Risankizumab, secukinumab, guselkumab, adalimumab, certolizumab, etanercept, ustekinumab, brodalumab, ixekizumab, tildrakizumab, infliximab, methotrexate, briakinumab, golimumab, and ADA (adalimumab). The primary outcomes assessed in the included studies were categorized into several domains. These domains included measures of disease severity such as PASI (Psoriasis Area and Severity Index) scores, ACR (American College of Rheumatology) scores for arthritis, and assessments for dactylitis, enthesitis, and nail involvement (Table 1).

**Table 1:** RCT included in the systematic review focusing on the outcomes of GRAPPA domain RCT: Randomized Controlled Trial, GRAPPA: Group for Research and Assessment of Psoriasis and Psoriatic Arthritis, PASI: Psoriasis Area and Severity Index Score, ACR: American College of Rheumatology score for arthritis. The data have been represented as a year of publication, number of participants (N), name of the drug used, dosage of the used drug (mg) and outcomes based on Psoriasis Area and Severity Index Score (PASI).

	Author	Year	Number	Drug	Dosage	Outcomes
1	Warren et al. [16]	2021	327	Risankizumab	150 mg	PASI75, PASI90, PASI100
				Secukinumab	300 mg	
2	Ferris et al. [17]	2020	78	Guselkumab	100 mg	PASI75, PASI90, PASI100
				Placebo		
3	McInnes et al. [18]	2020	853	Secukinumab	300 mg	ACR20, ACR50, ACR70, PASI75
				Adalimumab	40 mg	PASI90, PASI100, dactylitis assessment, enthesitis assessment
4	Mease et al. [19]	2020	741	Guselkumab	100 mg	ACR20, ACR50, ACR70, PASI75
				Placebo		PASI90, PASI100, dactylitis assessment, enthesitis assessment
5	Reich et al. [20]	2019	1048	Guselkumab	100 mg	PASI75, PASI90, PASI100
				Secukinumab	300 mg	
6	Ohtsuki et al. [21]	2019	171	Risankizumab	75 mg	PASI75, PASI90, PASI100
				Risankizumab	150 mg	
				Placebo		
7	Reich et al. [22]	2019	605	Risankizumab	150 mg	PASI75, PASI90, PASI100
				Adalimumab	40 mg	
8	Mease et al. [23]	2018	774	Secukinumab	150 mg	ACR20, ACR50, ACR70
				Secukinumab	300 mg	PASI75, PASI100, dactylitis assessment
				Placebo		
9	Gottlieb et al. [24] (CIMPASI-1)	2018	234	Certolizumab	200 mg	PASI90, PASI100
				Certolizumab	400 mg	
				Placebo		
	Gottlieb et al. [24] (CIMPASI-2)	2018	227	Certolizumab	200 mg	PASI90, PASI100
				Certolizumab	400 mg	
				Placebo		
10	Lebwohl et al. [25]	2018	559	Certolizumab	200 mg	PASI75, PASI90
				Certolizumab	400 mg	
				Etanercept	50 mg	
				Placebo		
11	Gordon et al. [26] (UltIMMa-1)	2018	506	Risankizumab	150 mg	PASI75, PASI90, PASI100
				Ustekinumab	45/90 mg	
				Placebo		
	Gordon et al. [26] (UltIMMa-2)	2018	491	Risankizumab	150 mg	PASI75, PASI90, PASI100
				Ustekinumab	45/90 mg	
				Placebo		
12	Reich et al. [27]	2018	198	Secukinumab	150 mg	PASI75, PASI90
				Secukinumab	300 mg	PASI100, nail assessment
				Placebo		
13	Bagel et al. [28]	2018	1102	Secukinumab	300 mg	PASI75, PASI90, PASI100
				Ustekinumab	45/90 mg	
14	Reich et al. [29] (reSURFACE 1)	2017	772	Tildrakizumab	100 mg	PASI75, PASI90, PASI100
				Tildrakizumab	200 mg	
				Placebo		
	Reich et al. [29] (reSURFACE 2)	2017	1,090	Tildrakizumab	100 mg	PASI75, PASI90, PASI100
				Tildrakizumab	200 mg	
				Etanercept	50 mg	
				Placebo		
15	Reich et al. [30]	2017	302	Ixekizumab	80 mg	PASI75, PASI90, PASI100
				Ustekinumab	45/90 mg	
16	Blauvelt et al. [31]	2017	837	Guselkumab	100 mg	PASI75, PASI90, PASI100, nail assessment
				Adalimumab	40 mg	
				Placebo		
17	Mease et al. [32]	2017	417	Ixekizumab	80 mg 2 w	ACR20, ACR50, PASI75
				Ixekizumab	80 mg 4 w	PASI90, PASI100, dactylitis assessment
				Adalimumab	40 mg	enthesitis assessment, nail assessment
				Placebo		
18	Gordon et al. [33]	2016	1,346	IXE	80 mg 2 w	PASI100, PASI90, PASI75
				IXE	80 mg 4 w	
				ETN	50 mg	
				PLB		
19	Papp et al. [34]	2016	661	Brodalumab	140 mg	PASI75, PASI90, PASI100
				Brodalumab	210 mg	
				Placebo		
20	Griffiths et al. [35] (UNCOVER-2 desgin)	2015	1,224	Ixekizumab	80 mg 2 w	PASI75, PASI90, PASI100
				Ixekizumab	80 mg 4 w	
				Etanercept	50 mg	
				Placebo		
	Griffiths et al. [35] (UNCOVER-3 design)	2015	1,346	Ixekizumab	80 mg 2 w	PASI75, PASI90, PASI100
				Ixekizumab	80 mg 4 w	
				Etanercept	50 mg	
				Placebo		
21	Lebwohl et al. [36] (AMAGINE-2 )	2015	1,831	Brodalumab	140 mg	PASI75, PASI100
				Brodalumab	210 mg	
				Ustekinumab	45/95 mg	
				Placebo		
	Lebwohl et al. [36] (AMAGINE-3)	2015	1,881	Brodalumab	140 mg	PASI75, PASI100
				Brodalumab	210 mg	
				Ustekinumab	45/95 mg	
				Placebo		
22	Thaçi et al [37]	2015	676	Secukinumab	300 mg	PASI75, PASI90, PASI100
				Ustekinumab	45/90 mg	
23	Langley et al. [38] (ERASURE study)	2014	738	Secukinumab	150 mg	PASI75, PASI90, PASI100
				Secukinumab	300 mg	
				Placebo		
	Langley et al. [38] (FIXTURE study)	2014	1,306	Secukinumab	150 mg	PASI75, PASI90, PASI100
				Secukinumab	300 mg	
				Etanercept	50 mg	
				Placebo		
24	Mease et al. [39]	2014	409	Certolizumab	200 mg	ACR20, ACR50, ACR70
				Certolizumab	400 mg	PASI50, PASI75, PASI90
				Placebo		Dactylitis assessment, enthesitis assessment, nail assessment
25	Baranauskaite et al. [40]	2012	115	Infliximab + Methotrexate	5 mg/kg	ACR20, ACR50, ACR70
				Methotrexate	15 mg	PASI75, dactylitis assessment, enthesitis assessment
26	Gottlieb et al. [41]	2012	478	Methotrexate + Etanercept	15 mg + 50 mg	PASI50, PASI75, PASI90
				Etanercept + placebo		
27	Barker et al. [42]	2011	868	Infliximab	5 mg/kg	PASI50, PASI75, PASI90
				Methotrexate	15 mg	
28	Gottlieb et al. [43]	2011	347	Briakinumab	200 mg	PASI75, PASI90, PASI100
				Etanercept	50 mg	
				Placebo		
29	Strober et al. [44]	2011	350	Briakinumab	200 mg	PASI75, PASI90, PASI100
				Etanercept	50 mg	
				Placebo		
30	Kavanaugh et al. [45]	2009	405	Golimumab	50 mg	ACR20, ACR50, ACR70
				Golimumab	100 mg	PASI50, PASI75, PASI90
				Placebo		dactylitis assessment enthesitis assessment, nail assessment
31	Leonardi et al. [46]	2008	766	Ustekinumab	45 mg	PASI50, PASI75, PASI90
				Ustekinumab	90 mg	
				Placebo		
32	Menter et al. [47]	2008	1212	Adalimumab	40 mg	PASI90, PASI100
				PLB		
33	Papp et al. [48]	2008	1,230	Ustekinumab	45 mg	PASI50, PASI75, PASI90
				Ustekinumab	90 mg	
				Placebo		
34	Tyring et al. [49]	2007	618	Etanercept	50 mg	PASI50, PASI75, PASI90
				Placebo		
35	Antoni et al. [50]	2005	200	Infliximab	5 mg/kg	ACR20, ACR50, ACR70, PASI50
				Placebo		PASI75, PASI90, PASI100, dactylitis assessment
36	Mease et al. [51]	2005	313	Adalimumab	40 mg	ACR20, ACR50, ACR70
				Placebo		PASI50, PASI75, PASI90
37	Reich et al. [52]	2005	378	Infliximab	5 mg/kg	PASI50, PASI75
				Placebo		PASI90, nail assessment

The study included a total of 30,023 participants, with approximately two-thirds of them being males (19,929 individuals, accounting for 66.37% of the total sample). The mean age of the participants was 45.76 years, with a standard deviation of 2.10. The age range varied from 40.1 to 53.3 years. The average duration of plaque psoriasis among the participants was 16.47 years, with a standard deviation of 4.04. The minimum duration reported was 2.8 years, while the maximum duration reached 21 years. The body surface area affected by psoriasis had a mean value of 26.87%, with a standard deviation of 5.06. The range for body surface area varied from 12% to 41.6%.

Among the participants, 15,507 individuals had their sPSA (severity of psoriasis) categorized. Of these, 8,773 patients (56.5%) were classified as having severe psoriasis (category 3), 6,671 patients (43.0%) were categorized as having very severe psoriasis (category 4 or above), and 63 patients (0.5%) had milder cases of psoriasis (below category 3).

The study also investigated the use of different medications for psoriasis treatment. The sample sizes ranged from 277 patients for briakinumab to 3,709 patients for secukinumab. The mean ages were generally similar across medications, ranging from 41.8 years for methotrexate to 48.65 years for Risankizumab. The percentage of male patients was also comparable, varying from 57.3% for infliximab to 75.7% for guselkumab. The number of studies analyzed per medication ranged from one trial for tildrakizumab, golimumab, and briakinumab to eight studies for etanercept and secukinumab. Placebo arms accounted for the largest pooled sample size of 5,542 patients across 27 studies (Table 2).

**Table 2:** General characteristics of the population and treatment sPGA: Static Physicians Global Assessment, N: Number of participants, N%: Percentage of the participants from the total participants in the study, NA: Not Available. The data have been represented as the name of the drug used, sample size, age (Year), gender (Male), average duration of plaque psoriasis (Year), sPGA category represents the score of psoriasis based on Static physician global assessment score which ranges from 0 (No signs of plaque psoriasis) to 4 (Dark, red erythematous psoriatic plaques), next to each score is the number of participants in the study and their percentage from the overall participants in that particular study, last column shows the percentage of the average of body area involved.

Author	Drug	Sample size	Age (Year) Mean	Gender (Male) Number (N%)	Duration of plaque psoriasis (years) Mean	sPGA category, n (%)			Body surface area (%), mean
						3 N (N%)	4 N (N%)	< 3 or missing. N (N%)	
Warren et al. [16]	Risankizumab	164	47·3	112 (68·3)	18·6	140 (85·4)	24 (14·6)	0	23·8
	Secukinumab	163	46·8	101 (62·0)	17·4	137 (84·0)	25 (15·3)	1 (0·6)	26·0
Ferris et al. [17]	Guselkumab	62	46.2	41 (66.1)	19.1	52 (83.9)	10 (16.1)	0	20.1
	Placebo	16	45.4	12 (75.0)	17.4	14 (87.5)	2 (12.5)	0	18.6
McInnes et al. [18]	Secukinumab	426	48·5	208 (49%)	5·1	215 (50%)	-	-	-
	Adalimumab	427	49·5	229 (54%)	5·7	202 (47%)	-	-	-
Mease et al. [19]	Guselkumab	493	45.4	271 (54.9%)	5·5	NA	NA	NA	18·2
	Placebo	246	46.3	117 (48%)	5·8	NA	NA	NA	17·1%
Reich et al. [20]	Guselkumab	534	46·3	365 (68%)	18·5	407 (76%)	127 (24%)	0	23·7
	Secukinumab	514	45·3	342 (67%)	18·3	391 (76%)	122 (24%)	1 (<1%)	24·5
Ohtsuki et al. [21]	Risankizumab (75 mg)	58	51.5	48 (83)	NA	NA	7 (12)	NA	41.6
	Risankizumab (150 mg)	55	53.3	50 (91)	NA	NA	9 (16)	NA	40.5
	Placebo	58	50.9	45 (78)	NA	NA	4 (7)	NA	33.2
Reich et al. [22]	Risankizumab	301	45·3	210 (70%)	NA	NA	NA	NA	26·5
	Adalimumab	304	47·0	212 (70%)	NA	NA	NA	NA	25·5
Mease et al. [23]	Secukinumab (300 mg)	222	48.9	108 (48.6)	6.7	NA	NA	NA	NA
	Secukinumab (150 mg)	220	48.4	111 (50.5)	6.7	NA	NA	NA	NA
	Placebo	332	49	161 (48.5)	6.6	NA	NA	NA	NA
Gottlieb et al. [24] (CIMPASI-1)	Certolizumab (200 mg)	95	44.5	67 (70.5)	16.6	62 (65.3)	33 (34.7)	NA	25.4
	Certolizumab (400)	88	43.6	60 (68.2)	18.4	65 (73.9)	23 (26.1)	NA	24.1
	Placebo	51	47.9	35 (68.6)	18.5	35 (68.6)	16 (31.4)	NA	26.1
Gottlieb et al. [24] (CIMPASI-2)	Certolizumab (200 mg)	91	46.7	58 (63.7)	18.8	66 (72.5)	25 (27.5)	NA	21.4
	Certolizumab (400)	87	46.4	43 (49.4)	18.6	61 (70.1)	26 (29.9)	NA	23.1
	Placebo	49	43.3	26 (53.1)	15.4	37 (75.5)	12 (24.5)	NA	20
Lebwohl et al. [25]	Certolizumab (200 mg)	165	46.7	113 (68.5)	19.5	114 (69.1)	51 (30.9)	NA	28.1
	Certolizumab (400 mg )	167	45.4	107 (64.1)	17.8	113 (67.7)	54 (32.3)	NA	27.6
	Etanercept	170	44.6	127 (74.7)	17.4	115 (67.6)	55 (32.4)	NA	27.5
	Placebo	57	46.5	34 (59.6)	18.9	40 (70.2	17 (29.8)	NA	24.3
Gordon et al. [26] (UltIMMa-1)	Risankizumab	304	48.3	212 (70%)	NA	256 (84%)	48 (16%)	NA	26.2
	Ustekinumab	100	46.5	70 (70%)	NA	85 (85%)	15 (15%)	NA	25.2
	Placebo	102	49.3	79 (77%)	NA	86 (84%)	16 (16%)	NA	27.9
Gordon et al. [26] (UltIMMa-2)	Risankizumab	294	46.2	203 (69%)	NA	228 (78%)	66 (22%)	NA	26.2
	Ustekinumab	99	48.6	66 (67%)	NA	81 (82%)	18 (18%)	NA	20.9
	Placebo	98	46.3	67 (68%)	NA	77 (79%)	21 (21%)	NA	23.9
Reich et al. [27]	Secukinumab (300 mg)	66	45.1	53 (80)	18	NA	NA	NA	28
	Secukinumab (150 mg)	67	43.5	55 (82)	20	NA	NA	NA	26.4
	Placebo	65	43.6	52 (80)	17.4	NA	NA	NA	25.8
Bagel et al. [28]	Secukinumab	550	45.4	356 (64.7)	16.8	NA	NA	NA	29.2
	Ustekinumab	552	45.3	376 (68.1)	17.3	NA	NA	NA	29.5
Reich et al. [29] (reSURFACE 1)	Tildrakizumab 200 mg	308	46·9	226 (73%)	NA	NA	NA	NA	30.9
	Tildrakizumab 100 mg	309	46·4	207 (67%)	NA	NA	NA	NA	29.7
	Placebo	155	47·9	100 (65%)	NA	NA	NA	NA	29.6
Reich et al. [29] (reSURFACE 2)	Tildrakizumab 200 mg	314	44·6	225 (72%)	NA	NA	NA	NA	31.8
	Tildrakizumab 100 mg	307	44·6	220 (72%)	NA	NA	NA	NA	34.2
	Etanercept	313	45·8	222 (71%)	NA	NA	NA	NA	31.6
	Placebo	156	46·4	112 (72%)	NA	NA	NA	NA	31.3
Reich et al. [30]	Ixekizumab	136	42.7	90 (66·2)	18	NA	NA	NA	26.7
	Ustekinumab	166	44·0	112 (67·5)	18.2	NA	NA	NA	27.5
Blauvelt et al. [31]	Guselkumab	329	43.9	240 (72.9)	17.9	252 (76.6)	77 (23.4)	3 (0.9)	28.3
	Adalimumab	334	42.9	249 (74.6)	17	241 (72.2)	90 (26.9)	0	28.6
	Placebo	174	44.9	119 (68.4)	17.6	131 (75.3)	43 (24.7)	0	25.8
Mease et al. [32]	Ixekizumab (once every 2 weeks)	103	49.8	48 (46.6)	17	NA	NA	NA	12
	Ixekizumab (once every 4 weeks)	107	49.1	45 (42.1)	16.5	NA	NA	NA	15.1
	Adalimumab	101	48.6	51 (50.5)	15.7	NA	NA	NA	14.8
	Placebo	106	50.6	48 (45.3)	16	NA	NA	NA	14.4
Gordon et al. [33]	Ixekizumab (once every 2 weeks)	386	46	254 (66.0)	18	NA	178 (46.2)	NA	28
	Ixekizumab (once every 4 weeks)	385	46	258 (66.8)	18	NA	177 (46.2)	NA	28
	Etanercept	382	46	269 (70.4)	18	NA	192 (50.3)	NA	28
	PLB	193	46	137 (71.0)	18	NA	101 (52.3)	NA	29
Papp et al. [34]	Brodalumab(140 mg)	219	46	162 (74)	19	129 (59)	94 (41)	0	27.4
	Brodalumab (210 mg)	222	46	161 (73)	20	121 (55)	97 (45)	0	25.1
	Placebo	220	47	161 (73)	21	114 (52)	106 (48)	0	26.9
Griffiths et al. [35] (UNCOVER-2 desgin)	Ixekizumab (once every 2 weeks)	351	45	221 (63%)	19	NA	173 (49%)	NA	25
	Ixekizumab (once every 4 weeks)	347	45	244 (70%)	19	NA	181 (52%)	NA	27
	Etanercept	358	45	236 (66%)	19	NA	172 (48%)	NA	25
	Placebo	168	45	120 (71%)	19	NA	82 (49%)	NA	27
Griffiths et al. [35] (UNCOVER-3 design)	Ixekizumab (once every 2 weeks)	385	46	254 (66%)	18	NA	178 (46%)	NA	28
	Ixekizumab (once every 4 weeks)	386	46	258 (67%)	18	NA	177 (46%)	NA	28
	Etanercept	382	46	269 (70%)	18	NA	192 (50%)	NA	28
	Placebo	193	46	137 (71%)	18	NA	101 (52%)	NA	29
Lebwohl et al. [36] (AMAGINE-2 )	Brodalumab(140 mg)	610	45	413 (68)	19	358 (59)	52	NA	27
	Brodalumab (210 mg)	612	45	421 (69)	19	316 (52)	296 (48)	NA	26
	Ustekinumab	300	45	205 (68)	19	153 (51)	147 (49)	NA	27
	Placebo	309	44	219 (71)	18	167 (54)	142 (46)	NA	28
Lebwohl et al. [36] (AMAGINE-3)	Brodalumab	629	45	437 (70)	17	412 (66)	217 (34)	NA	29
	Brodalumab	624	45	431 (69)	18	373 (60)	251 (40)	NA	28
	Ustekinumab	313	45	212 (68)	18	192 (61)	121 (39)	NA	28
	Placebo	315	44	208 (66)	18	192 (61)	123 (39)	NA	28
Thaçi et al. [37]	Secukinumab	337	45.2	229 (68.0)	19.6	NA	130 (38.6)	NA	32.6
	Ustekinumab	339	44.6	252 (74.3)	16.1	NA	125 (36.9)	NA	32
Langley et al. [38] (ERASURE study)	Secukinumab (300 mg)	245	44.9	169 (69.0)	17.4	154 (62.9)	91 (37.1)	NA	32.8
	Secukinumab (150 mg)	245	44.9	168 (68.6)	17.5	161 (65.7)	84 (34.3)	NA	33.3
	Placebo	248	45.4	172 (69.4)	17.3	151 (60.9)	97 (39.1)	NA	29.7
Langley et al. [38] (FIXTURE study)	Secukinumab (300 mg)	327	44.5	224 (68.5)	15.8	203 (62.1)	124 (37.9)	NA	34.3
	Secukinumab (150 mg)	327	45.4	236 (72.2)	17.3	206 (63 )	121 (37)	NA	34.5
	Etanercept	326	43.8	232 (71.2)	16.4	195 (59.8)	131 (40.2)	NA	33.6
	Placebo	326	44.1	237 (72.7)	16.6	202 (62)	124 (38.0)	NA	35.2
Mease et al. [39]	Certolizumab (200 mg Q2W)	138	48.2	64 (46.4)	9.6	NA	NA	NA	NA
	Certolizumab (400 mg Q4W)	135	47.1	62 (45.9)	8.1	NA	NA	NA	NA
	Placebo	136	47.3	57 (41.9)	7.9	NA	NA	NA	NA
Baranauskaite et al. [40]	Infliximab + Methotrexate	56	40.1	27 (48.2)	2.8	NA	NA	NA	NA
	Methotrexate	54	42.3	33 (61.1)	3.7	NA	NA	NA	NA
Gottlieb et al. [41]	Methotrexate + Etanercept	239	43	153 (64·0)	17.9	138 (57·7)	69 (28.9)	32 (13.4)	24.4
	Etanercept + placebo	239	45.2	167 (69·9)	16.9	139 (58·2)	74(31)	26(10.8)	24.2
Barker et al. [42]	Infliximab	653	44.1	438 (67)	NA	NA	NA	NA	31.9
	Methotrexate	215	41.9	148 (69)	NA	NA	NA	NA	31
Gottlieb et al. [43]	Briakinumab	138	43.6	89 (64.5)	16.1	77 (55.8)	61 (44.2)	0	23.6
	Etanercept	141	43.1	98 (69.5)	17	72 (51.1)	69 (48.9)	0	24.1
	Placebo	68	44	47 (69.1)	19.1	42 (61.8)	26 (38.2)	0	23.8
Strober et al. [44]	Briakinumab	139	44.9	93 (66.9)	16.3	63 (45.3)	38 (44.7)	NA	24.9
	Etanercept	139	45.2	85 (61.2)	15.2	69 (49.6)	70 (50.4)	NA	24.7
	Placebo	72	45	46 (63.9)	15.5	34 (47.2)	76 (52.8)	NA	22.1
Kavanaugh et al. [45]	Golimumab (50 mg)	146	45.7	89 (61)	7.2	NA	NA	NA	16.2
	Golimumab (100 mg)	146	48.2	86 (59)	7.7	NA	NA	NA	17.7
	Placebo	113	47	69 (61)	7.6	NA	NA	NA	14.7
Leonardi et al. [46]	Ustekinumab (45 mg)	255	44.8	175 (68·6%)	19.7	NA	NA	NA	27.2
	Ustekinumab (90 mg)	256	46.2	173 (67·6%)	19.6	NA	NA	NA	25.2
	Placebo	255	44.8	183 (71·8%)	20.4	NA	NA	NA	27.7
Menter et al. [47]	Adalimumab	814	44.1	546 (67.1)	18.1	417 (51.2)	397 (48.8)	NA	25.8
	Placebo	398	45.4	257 (64.6)	18.4	220 (55.3)	178 (44.7)	NA	25.6
Papp et al. [48]	Ustekinumab (45 mg)	409	45.1	283 (69·2%)	19.3	NA	NA	NA	25.9
	Ustekinumab(90 mg)	411	46.4	274 (66·7%)	20.3	NA	NA	NA	27.1
	Placebo	410	47	283 (69·0%)	20.8	NA	NA	NA	26.1
Tyring et al. [49]	Etanercept	311	45.8	203 (65.3)	20.2	NA	NA	NA	27.2
	Placebo	307	45.5	215 (70.0)	19.7	NA	NA	NA	27.2
Antoni et al. [50]	Infliximab	100	47.1	71 (71)	8.4	NA	NA	NA	NA
	Placebo	100	46.5	51 (51)	7.5	NA	NA	NA	NA
Mease et al. [51]	Adalimumab	151	48.6	85 (56.3)	9.8	NA	NA	NA	NA
	Placebo	162	49.2	89 (54.9)	9.2	NA	NA	NA	NA
Reich et al. [52]	Infliximab	301	42.6	207 (69)	19.1	NA	NA	NA	34.1
	Placebo	77	43.8	61 (79)	17.3	NA	NA	NA	33.5

The results of the study revealed significant differences between the medications and placebo, as well as variations among the different medications themselves. Among the various medications, guselkumab demonstrated the highest efficacy, with PASI75, PASI90, and PASI100 improvement rates of 89.63%, 72.7%, and 48.47% respectively. Following closely behind was ixekizumab, exhibiting impressive improvement rates of 81.33%, 71.53%, and 37.83% respectively for PASI75, PASI90, and PASI100. Risankizumab and briakinumab also showed notable efficacy, with PASI scores of 84.5%, 66.83%, and 34.8% for Risankizumab, and 83.14%, 65.94%, and 34.62% for briakinumab. Adalimumab, certolizumab, secukinumab, ustekinumab, and methotrexate also exhibited moderate effectiveness in improving psoriasis symptoms, although with varying degrees. These medications demonstrated PASI improvement rates ranging from 50% to 73.8% for PASI75, 37.65% to 50.73% for PASI90, and 13.15% to 24.28% for PASI100. On the other hand, etanercept, golimumab (50 mg dosage), and TIL 100 mg showed relatively lower efficacy compared to other medications. Etanercept resulted in PASI75, PASI90, and PASI100 improvement rates of 43.24%, 17%, and 4.48%, respectively, while golimumab and TIL 100 mg exhibited results of 40.3%, 20.8%, and 62.5%, 36.9%, 13.15%, respectively. Comparing the medications to the placebo group, all the biological medications showed significantly higher improvement rates across the PASI scores. The placebo group had minimal improvements, with PASI75, PASI90, and PASI100 rates of 5.76%, 1.8%, and 0.45% respectively (Table 3).

**Table 3:** sPASI Improvements in patients with psoriasis skin sPASI: Simplified Psoriasis Severity Index, PASI: Psoriasis Area and Severity Index Score, N: Number of the participants, %: Percentage of the participants from the overall participants. The data have been represented as weeks of treatment, name of the drug used, PASI100 (Completely clear skin), PASI90 (Clear to almost clear skin), and PASI75 (75% reduction of severity from the baseline)

			PASI100	PASI90	PASI75	
Study	Weeks	Drug	n/total (%)	n/total (%)	n/total (%)	
Warren et al. [16]	16 weeks	Risankizumab	44/164 (26.9)	74/164 (45.1)	92/164 (56.1)	
	16 weeks	Secukinumab	34/163 (20.9)	66/163 (40.5)	80/163 (49.1)	
		Significance	Significant	Significant	Significant	
Ferris et al. [17]	16 weeks	Guselkumab	31/62 (50.0)	47/62 (75.8)	55/62 (88.7)	
	16 weeks	Placebo	0/16 (0)	0/16 (0)	0/16 (0)	
		Significance	Significant	Significant	Significant	
McInnes et al. [18]	52 weeks	Secukinumab	99/215 (46)	140/215 (54)	170/215 (79)	
	52 weeks	Adalimumab	61/202 (30)	87/202 (43)	123/202 (61)	
		Significance	Significant	Significant	Significant	
Mease et al. [19]	16 weeks	Adalimumab	132/283 (46.6)	158/283 (55.8)	195/238 (68.9)	
	16 weeks	ixekizumab	170/283 (60.1)	203/283 (71.7)	227/283 (80.2)	
		Significance	Significant	Significant	Significant	
Reich et al. [20]	12 weeks	GUSELKUMAB	311/534 (58)	369/534 (69)	477/534 (89)	
	12 weeks	Secukinumab	249/514 (48)	391/514 (76)	471/514 (92)	
		Significance	NA	NA	NA	
Ohtsuki et al. [21]	16 weeks	Risankizumab75 mg	13/58 (22.4)	–	52/58 (89.8)	
						16 weeks	Risankizumab 150 mg	18/55 (32.7)	–	52/55 (94.5)	
	16 weeks	Placebo	0/0	–	5/58 (8.6)	
		Significance	Significant		Significant	
	Reich et al. [22]	16 weeks	Risankizumab 150 mg	120/301 (40)	218/301 (72)	237/301 (91)	
16 weeks		Adalimumab	70/304 (23)	144/304 (47)	218/304 (72)	
		Significance	Significant	Significant	Significant	
Mease et al. [23]	16 weeks	Placebo	–	31/332 (9.3)	40/332 (12.3)	
	16 weeks	Secukinumab 150 mg	–	81/220 (36.8)	132/220 (60.0)	
	16 weeks	Secukinumab 300 mg	–	119/222 (53.6)	155/222 (70.0)	
		Significance		Significant	Significant	
Gottlieb et al. [24]	16 weeks	Placebo	0/51 (0.0)	0/51 (0.0)	3/51 (6.5)	
	16 weeks	certolizumab 200 mg	13/95 (13.7)	34/95 (35.8)	63/95 (66.3)	
		Significance	Significant	Significant	Significant	
Gottlieb et al. [24]	16 weeks	Placebo	1/49 (1.8)	2/49 (2.2)	6/49 (11.6)	
	16 weeks	certolizumab 200 mg	14/91 (15.4)	48/91 (52.6)	74/92 (81.4)	
		Significance	Significant	Significant	Significant	
Lebwohl et al. [25]	16 weeks	Placebo	–	5/57 (0.0)	3/57 (5.3)	
	16 weeks	certolizumab 200 mg	–	66/165 (40.0)	113/165 (68.5)	
		Significance		Significant	Significant	
Gordon et al. [26]	12 weeks	Placebo	0/102 (0.0)	2/102 (2.0)	10/102 (9.8)	
	12 weeks	ustekinumab*	12/100 (12.0)	42/100 (42.0)	70/100 (70)	
	12 weeks	Risankizumab	109/304 (35.9)	229/304 (75.3)	264/304 (86.8)	
		Significance	Significant	Significant	Significant	
Gordon et al. [26]	12 weeks	Placebo	2/98 (2.0)	2/98 (2.0)	8/98 (8.1)	
	12 weeks	ustekinumab	24/99 (24.2)	47/99 (47.5)	69/99 (69.7)	
	12 weeks	Risankizumab	149/294 (50.7)	220/294 (74.9)	261/294 (88.8)	
		Significance	Significant	Significant	Significant	
Reich et al. [27]	16 weeks	Placebo	0/65 (0.0)	1/65 (1.5)	3/65 (4.6)	
	16 weeks	Secukinumab 300 mg	22/66 (33.3)	48/66 (72.7)	56/66 (84.8)	
		Significance		Significant	Significant	
Bagel et al. [28]	16 weeks	Secukinumab	249/550 (45.3)	421/550 (76.6)	504/550 (91.7)	
	16 weeks	ustekinumab	147/552 (26.7)	299/552 (54.1)	440/552 (79.8)	
		Significance	Significant	Significant	Significant	
Reich et al. [29]	12 weeks	Placebo	2/154 (1.3)	4/154 (3.0)	9/154 (5.8)	
	12 weeks	tildrakizumab 100 mg	43/309 (13.9)	107/309 (35.0)	197/309 (63.8)	
		Significance	Significant	Significant	Significant	
Reich et al. [29]	12 weeks	Placebo	0/156 (0.0)	2/156 (1.3)	9/156 (5.8)	
	12 weeks	tildrakizumab 100 mg*	38/307 (12.4)	119/307 (38.8)	188/307 (61.2)	
	12 weeks	Etanercept	15/313 (4.8)	67/313 (21.4)	151/313 (48.2)	
		Significance	Significant	Significant	Significant	
Reich et al. [30]	12 weeks	ixekizumab	49/136 (36.0)	99/136 (72.8)	120/136 (88.2)	
	12 weeks	ustekinumab	24/166 (14.5)	70/166 (42, 2)	114/166 (68.7)	
		Significance	Significant	Significant	Significant	
Blauvelt et al. [31]	16 weeks	Placebo	1/174(0.6)	5/174 (2.9)	10/174 (5.7)	
	16 weeks	GUSELKUMAB*	123/329 (37.4)	241/329 (73, 3)	300/329 (91.2)	
	16 weeks	Adalimumab	57/334 (17.4)	166/334 (49.7)	244/334 (73.1)	
		Significance	Significant	Significant	Significant	
Mease et al. [32]	12 weeks	Placebo	1/67 (1.5)	1/67 (1.5)	5/67 (7.5)	
	12 weeks	ixekizumab Q4W*	23/73 (31.5)	38/73 (52.0)	55/73 (75.3)	
	12 weeks	Adalimumab	10/68 (14.7)	15/68 (22.1)	23/68 (33.8)	
		Significance	Significant	Significant	Significant	
Gordon et al. [33]	12 weeks	Placebo	0/431 (0.0)	7/431 (1.7)	17/431 (3.9)	
	12 weeks	ixekizumab Q4W	145/432 (33.6)	279/432 (64.6)	357/432 (82.6)	
		Significance	Significant	Significant	Significant	
Papp et al. [34]	12 weeks	Placebo	1/220 (0.5)	2/220 (0.9)	6/220 (2.7)	
	12 weeks	brodalumab	93/222 (41.9)	156/220 (70.9)	185/222 (83.3)	
		Significance	Significant	Significant	Significant	
Griffiths et al. [35]	12 weeks	Placebo	1/168 (0.6)	1/168 (0.6)	4/168 (2.4)	
	12 weeks	ixekizumab Q4W*	107/347 (30.8)	267/347 (76.9)	269/347 (77.5)	
	12 weeks	Etanercept	19/358 (5.3)	67/358 (18.7)	149/358 (41.6)	
		Significance	Significant	Significant	Significant	
Griffiths et al. [35]	12 weeks	Placebo	0/193 (0.0)	6/193 (3.1)	14/193 (7.2)	
	12 weeks	ixekizumab*	135/386 (35.0)	352/386 (91.2)	325/386 (84.2)	
	12 weeks	Etanercept	19/358 (5.3)	98/382 (25.6)	201/382 (52.6)	
		Significance	Significant	Significant	Significant	
Lebwohl et al. [36]	12 weeks	Placebo	2/309 (0.6)	12/309 (3.9)	25/309 (8.1)	
	12 weeks	ustekinumab	65/300 (21.7)	141/300 (47.0)	210/300 (70.0)	
	12 weeks	brodalumab 210 mg	272/612 (44.4)	428/612 (69.9)	528/612 (86.3)	
		Significance	Significant	Significant	Significant	
Lebwohl et al. [36]	12 weeks	Placebo	1/315 (0.3)	6/315 (1.9)	19/315 (6.0)	
	12 weeks	ustekinumab	58/313 (18.5)	141/313 (45.0)	217/313 (69.3)	
	12 weeks	brodalumab	229/624 (36.7)	430/624 (68.9)	531/624 (85.1)	
		Significance	Significant	Significant	Significant	
Thaci et al. [37]	12 weeks	SEC	148/334 (44.3)	264/334 (79.0)	311/334 (93.1)	
	12 weeks	ustekinumab	130/334 (38.9)	277/334 (82.9)	277/334 (82.9)	
		Significance	Significant	Significant	Significant	
Langley et al. [38]	12 weeks	Placebo	2/246 (0.8)	3/246 (1.2)	11/246 (4.5)	
	12 weeks	Secukinumab 300 mg	70/245 (26.6)	145/245 (59.2)	200/245 (81.6)	
		Significance	Significant	Significant	Significant	
Langley et al. [38]	12 weeks	Placebo	0/324 (0.0)	5/324 (1.5)	16/324 (4.9)	
	12 weeks	Secukinumab 300 mg	78/323 (24.1)	175/323 (54.1)	249/323 (77.0)	
	12 weeks	Etanercept	14/323 (4.3)	67/323 (20.7)	142/323 (44.0)	
		Significance	Significant	Significant	Significant	
Mease et al. [39]	12 weeks	Placebo	–	4/86 (4.7)	12/86 (13.9)	
	12 weeks	certolizumab 200 mg	–	20/90 (22.2)	42/90 (46.7)	
		Significance		Significant	Significant	
Baranauskaite et al. [40]	16 weeks	Methotrexate	–	–	19/35 (54.3%)	
	16 weeks	Methotrexate+ infliximab	–	–	33/34 (97.1%)	
		Significance	–	–	Significant	
Gottlieb et al. [41]	24 weeks	Methotrexate + etanercept	–	–	184/239 (77.3%)	
	24 weeks	etanercept+ Placebo	–	–	144/239 (60·3%)	
		Significance	–	–	Significant	
Barker et al. [42]	16 weeks	Methotrexate	–	41/216 (19.0)	90/216 (41.7)	
	16 weeks	Infliximab	–	356/656 (54.2)	508/656 (77.4)	
		Significance		Significant	Significant	
Gottlieb et al. [43]	12 weeks	Placebo	0/68 (0.0)	1/68 (1.5)	5/68 (7.4)	
	12 weeks	briakinumab	39/138 (28.3)	83/138 (60.0)	112/138 (81.0)	
	12 weeks	Etanercept	5/141 (3.6)	18/141 (12.7)	78/141 (55.0)	
		Significance	Significant	Significant	Significant	
Strober et al. [44]	12 weeks	Placebo	0/72 (0.0)	3/72 (4.2)	5/72 (6.9)	
	12 weeks	briakinumab	30/139 (21.9)	83/139 (60)	111/139 (80.0)	
	12 weeks	Etanercept	5/139 (3.6)	18/139 (13.0)	40/139 (28.8)	
		Significance	Significant	Significant	Significant	
Kavanaugh et al. [45]	14 weeks	Placebo	–	0/73 (0.0)	2/79 (2.5)	
	14 weeks	golimumab 50 mg	–	22/106 (20.8)	44/109 (40.3)	
		Significance		Significant	Significant	
Leonardi et al. [46]	12 weeks	Placebo	0/255 (0.0)	5/255 (2.0)	5/255 (2.0)	
	12 weeks	ustekinumab 45 mg	32/255 (12.5)	106/255 (41.6)	171/255 (67.0)	
		Significance	Significant	Significant	Significant	
Menter et al. [47]	12 weeks	Placebo	4/398 (1.0)	8/398 (2.0)	20/398 (5.0)	
	12 weeks	Adalimumab	114/814 (14.0)	301/814 (37.0)	554/814 (68.1)	
		Significance	Significant	Significant	Significant	
Papp et al. [48]	12 weeks	Placebo	0/410 (0.0)	3/410 (0.7)	15/410 (3.7)	
	12 weeks	ustekinumab 45 mg	74/409 (18.1)	173/409 (42.3)	273/409 (66.5)	
		Significance	Significant	Significant	Significant	
Tyring et al. [49]	12 weeks	Placebo	–	1/292 (0.3)	5/292 (1.7)	
	12 weeks	Etanercept	–	21/305 (6.9)	47/305 (15.4)	
		Significance		Significant	Significant	
Antoni et al. [50]	14 weeks	Placebo	–	0/87 (0.0)	1/87 (1.0)	
	14 weeks	Infliximab	–	34/87 (41.0)	55/87 (64.0)	
		Significance		Significant	Significant	
Mease et al. [51]	12 weeks	Placebo	–	0/69 (0.0)	4/69 (5.8)	
	12 weeks	Adalimumab	–	30/69 (43.5)	49/69 (71.0)	
		Significance		Significant	Significant	
Reich et al. [52]	10 weeks	Placebo	–	1/77 (1.0)	2/77 (3.0)	
	10 weeks	Infliximab	–	172/301 (57.0)	242/301 (80.0)	
		Significance		Significant	Significant	

Discussion

From among the several drugs that were evaluated, guselkumab consistently exhibited the most notable rates of improvement across all three PASI categories: 89.63%, 72.7%, and 48.47% for PASI75, PASI90, and PASI100, respectively. Consistent with prior research, the effectiveness of Guselkumab in the treatment of psoriasis certifies its status as a very successful therapeutic alternative [53-55]. Ixekizumab had noteworthy effectiveness as well, as seen by improvement rates of 81.33%, 71.53%, and 37.83%, respectively, on the PASI75, PASI90, and PASI100, respectively. The findings of this study provide further support for the notion that ixekizumab is an effective treatment for psoriasis [56].

Both briakinumab and Risankizumab exhibited significant efficacy, as evidenced by the considerable rates of improvement observed in the PASI scores. Briakinumab demonstrated PASI scores of 34.62%, 63.14%, and 66.94%, whereas Risankizumab demonstrated PASI75, PASI90, and PASI100 scores of 84.5%, 66.83%, and 34.8%, respectively. The results underscore the efficacy of these pharmaceuticals in mitigating the symptoms associated with psoriasis.

Our results are similar to many previous studies that showed that the new biologic medicines, including Risankizumab [26], guselkumab [20,31,57,58], ixekizumab [59-62], and brodalumab [36,63], have proven high efficacy in patients with moderate-to-severe psoriasis. In the respective clinical trials, approximately 70%-80% of patients attained a reduction in the Psoriasis Area Severity Index (PASI) score of 90% or above within 16 weeks of therapy initiation (PASI 90) [64]. At 52 weeks, the proportion decreased to between 80% and 90%. PASI 100 was between 50% and 60% at 52 weeks. The biologic medicines exhibited significant efficacy [64].

Adalimumab, certolizumab, secukinumab, ustekinumab, and methotrexate were among the additional drugs that exhibited a modest degree of effectiveness in ameliorating symptoms associated with psoriasis. The observed variations in improvement rates among various drugs underscore the significance of taking into account the unique qualities and preferences of each patient when determining the most suitable course of treatment. It is noteworthy that although the improvement rates of these drugs may be comparatively lower than those of ixekizumab and guselkumab, they nonetheless provide substantial advantages in the management of psoriasis.

Conversely, the effectiveness of etanercept, golimumab (at a dosage of 50 mg), and TIL 100 mg was comparatively diminished in comparison to the aforementioned drugs. Etanercept induced the following percentage improvements in PASI75, PASI90, and PASI100: 43.24%, 17%, and 4.48%, respectively. The administration of 50 mg of golimumab resulted in 40.3% and 20.8% improvement rates for PASI75 and PASI90, respectively. Similarly, 100 mg of TIL produced improvement rates of 62.5%, 36.9%, and 13.15% for PASI75, PASI90, and PASI100, respectively. Patients whose responses to these drugs are inadequate may benefit more from alternate treatment modalities, according to these results.

Brodalumab demonstrated a significantly higher level of efficacy compared to secukinumab, ustekinumab, and etanercept, as evidenced by four 52-week RCTs. Similarly, secukinumab demonstrated more efficacy than ustekinumab, and both agents beat etanercept. The results obtained from thirteen supplementary trials and four additional therapeutic interventions (ixekizumab, apremilast, infliximab, and brodalumab) demonstrated that brodalumab exhibited the highest efficacy, followed by ustekinumab, infliximab, and ixekizumab. It was expected that etanercept would have the least lasting effect. At week 52, brodalumab was associated with a higher likelihood of prolonged PASI response, including complete clearance, in comparison to comparable medications. Furthermore, Sawyer et al. [65] did a network meta-analysis comprising 34,816 patients and 77 studies. The effectiveness of brodalumab, ixekizumab, secukinumab, guselkumab, and Risankizumab in the treatment of plaque psoriasis was shown to be superior to that of ustekinumab, tildrakizumab, all TNF-α inhibitors, non-biologic systemic medicines, as demonstrated by the researchers. Furthermore, it was observed that brodalumab, ixekizumab, and Risankizumab exhibited greater efficacy than secukinumab, however not by a substantial margin compared to guselkumab. In terms of PASI 90 and PASI 100 response, brodalumab, ixekizumab, guselkumab, and Risankizumab shown the most substantial improvements. According to a meta-analysis of 140 studies conducted by Shidian et al. [66], the percentage of patients attained by ixekizumab, secukinumab, bimekizumab, brodalumab, Risankizumab, and guselkumab with PASI 90 demonstrated that these agents were more effective than ustekinumab, adalimumab, certolizumab, and etanercept. Furthermore, adalimumab and ustekinumab had a higher degree of efficacy compared to certolizumab and etanercept. A comparison between the biological drugs and the placebo group provides more evidence of the biological therapies' better efficacy. The improvement rates of all biological drugs assessed on the PASI were found to be significantly greater in comparison to the placebo group. This underscores the significance of regarding these drugs as the benchmark for the management of psoriasis.

An optimal treatment regimen for a patient with psoriasis should consist of a solitary medication that exhibits efficacy across all indications. The study's exhaustive literature review offers significant insights into the effectiveness of several biological drugs in the treatment of psoriasis. Consistent with other investigations, the results validate the concept that briakinumab, ixekizumab, guselkumab, and ixekizumab are exceedingly efficacious therapeutic alternatives. Furthermore, methotrexate, adalimumab, certolizumab, secukinumab, and ustekinumab exhibit a moderate degree of efficacy in the management of symptoms associated with psoriasis.

By giving a complete review of the efficacy of various drugs, the results of this study contribute to the current body of knowledge on psoriasis treatment. However, a few limitations should be taken into account. The study initially utilized data obtained from randomised controlled trials, which might not comprehensively represent treatment outcomes in the real world. A more positive response to treatment may be observed in the controlled trial setting as opposed to ordinary clinical practice.

## Conclusions

The systematic review assessed the performance of several biological drugs in the treatment of psoriasis offers significant insights into the treatment's success. Adalimumab, certolizumab, secukinumab, ustekinumab, and methotrexate had moderate efficacy, whereas guselkumab, ixekizumab, Risankizumab, and briakinumab appeared as exceptionally successful alternatives. Clinicians can utilize these findings as a guide for determining which treatment is most suitable for specific patients. When making treatment options, it is essential to evaluate patient characteristics, treatment objectives, and potential adverse effects. Additional investigation into the long-term effects of various drugs and comparative analyses of their efficacy is necessary in order to advance our knowledge of psoriasis care.
